# The Atlanta College of Physicians and Surgeons

**Published:** 1905-11

**Authors:** 


### EDITORIAL NOTES AND COMMENTS.

The Business office of THE JOURNAL-RECORD is No. 1007-1008 Century Building.

The Editorial office is 318-319-320 The Prudential.

Address all Business communications to Dr. M. B. Hutchins, Mgr.

Make remittances payable to THE ATLANTA JOURNAL-RECORD OF MEDICINE.

On matters pertaining to the Editorial and Original Communications address Dr. Bernard Wolff, Atlanta.

Reprints of original articles will be furnished at cost price. Requests for the same should always be made *on the manuscript*.

We will present, post-paid, on request, to each contributor of an original article, twenty (20) marked copies of THE JOURNAL-RECORD containing such article.

### THE ATLANTA COLLEGE OF PHYSICIANS AND SURGEONS.

THE fifty-fifth annual session of the College of Physicians and Surgeons begun October 4.

Speeches were made at the opening exercises by Drs. A. W. Calhoun, Nicolson, Cooper and Elkin. The school was shown to be in an exceedingly stable condition, and the prospects for the ensuing year were unequalled in the history of the institution. A new and in every way modern and thoroughly equipped building was promised for the near future. The structure is to cost $100,000, a large part of which has already been subscribed, Dr. Calhoun heading the list with $10,000. The plans are in the hands of the architect and will soon be presented to the contractors for estimates on the building.

The following changes have been effected in the administrative and teaching corps of the faculty: Dr. W. S. Elkin has been elected Dean; Dr. S. T. Barnett, Registrar; Mr. C. F. Carlton appointed Clerk of the college.

The Chair of the Principles and Practice of Medicine, owing to the withdrawal from the faculty and from the school of Dr. W. S. Kendrick, has been subdivided. Dr. J. B. Baird will fill the chair, with Dr. C. G. Giddings as Clinical Professor of Medicine, and Dr. Cyrus W. Strickler Adjutant to the Chair. Dr. E. Bates Block has been named Clinical Professor of Diseases of the Mind and Nervous System; Dr. W. S. Goldsmith, Clinical Professor of Genito-Urinary Diseases and Surgery; Dr. Michael Hoke, Clinical Professor of Orthpedic Surgery; Dr. Bernard Wolff, Clinical Professor of Dermatology.

The following is a full list of professors and instructors:

A. W. Calhoun, M.D., LL.D., Professor of Diseases of the Eye, Ear, Nose and Throat, and of Clinical Opthalmology, Otology and Laryngology, President of the Faculty. (Oculist and Aurist of Grady Hospital and Wesley Memorial Hospital.)

William Perrin Nicolson, M.D., Professor of Anatomy and Clinical Surgery. (Surgeon to Grady Hospital and to Wesley Memorial Hospital.)

J. S. Todd, M.D., Professor of Materia Medica and Therapeutics. (Physician to Wesley Memorial Hospital.)

William Simpson Elkin, A.B., M.D., Professor of Operative and Clinical Surgery, Dean of Faculty. (Surgeon to Grady Hospital.)

Louis H. Jones, A.M., M.D., Professor of General and Medical Chemistry and Medical Jurisprudence.

W. F. Westmoreland, M.D., Professor of Principles and Practice of Surgery and Clinical Surgery.

Floyd Wilcox McRae, M.D., Professor of Gastro-Intestinal, Rectal and Clinical Surgery. (Surgeon to Grady Hospital.)

Hunter P. Cooper, M.D., Professor of Obstetrics and Gynecology. (Gynecologist to Wesley Memorial Hospital.)

J. Clarence Johnson, M.D., Professor Physiology and Diseases of the Digestive System.

H. F. Harris, M.D., Professor of Pathology, Bacteriology and Pathological Anatomy. (Physician and Pathologist to Wesley Memorial Hospital.)

Dunbar Roy, M.D., Clinical Professor of Diseases of the Eye, Ear, Nose and Throat. (Oculist and Aurist to Grady Hospital.)

John G. Earnest, M.D., Clinical Professor of Gynecology. (Gynecologist to Grady Hospital and to Presbyterian Hospital.)

James B. Baird, M.D., Professor of Principles and Practice of Medicine. (Physician to Grady Hospital.)

Charles G. Giddings, M.D., Professor of Clinical Medicine. (Surgeon to Grady Hospital.)

W. S. Goldsmith, M.D., Professor of Genito-Urinary Diseases and Clinical Surgery. (Surgeon to Tabernacle Infirmary.)

Cyrus W. Strickler, M.D., Associate Professor of Principles and Practice of Medicine. (Surgeon to Presbyterian Hospital.)

E. Bates Block, M.D., Professor of Mental and Nervous Diseases. (Physician to Presbyterian Hospital and to Tabernacle Infirmary.)

Bernard Wolff, M.D., Clinical Professor of Skin Diseases.

Michael F. Hoke, M.D., Clinical Professor of Orthopedic Surgery.

S. T. Barnett, M.D., Registrar Assistant to Chair of Clinical Gynecology. (Surgeon to Presbyterian Hospital.)

T. V. Hubbard, M.D., Assistant to Chair of Materia Medica and Therapeutics.

C. E. Boynton, A.B.. M.D., Lecturer on Diseases of Children. (Physician to Wesley Memorial Hospital.)

Claude A. Smith, M.D., Assistant to Chair of Pathology and Bacteriology. (Pathologist to Grady Hospital and to Tabernacle Infirmary.)

A. L. Fowler, M.D., Assistant to Chair of Principles and Practice of Surgery. (Physician to United States prison.)

W. B. Armstrong, M.D., Associate Demonstrator of Anatomy.

J. H. Johns, M.D., Associate Demonstrator of Anatomy.

L. S. Hardin, M.D., Assistant to Chair of Clinical Surgery.

Fred G. Hodson, M.D., Assistant to Chair of Obstetrics and Gynecology.

W. A. Selman, B.S., M.D., Lecturer on Osteology.

Hugh M. Lokey, M.D., Assistant to Chair of Diseases of Eye, Ear, Nose and Throat. (Oculist and Aurist to Wesley Memorial Hospital.)

W. E. Person, M.D., Lecturer on Minor Surgery and Bandaging.

